# Supplementary material for: Patterns of Heterochromatin Transitions Linked to Changes in the Expression of Plasmodium falciparum Clonally Variant Genes
Source: Microbiol Spectr. 2022 Dec 14;11(1):e03049-22. doi: 10.1128/spectrum.03049-22 (PMC9927496; doi:10.1128/spectrum.03049-22)
Supplement: Supplemental file 1 — Fig. S1 to S7. Download spectrum.03049-22-s0001.pdf, PDF file, 2.4 MB [file spectrum.03049-22-s0001.pdf]

SUPPLEMENTARY FIGURES

Patterns of heterochromatin transitions linked to changes in the expression of *Plasmodium falciparum* clonally variant genes

Lucas Michel-Todó, Cristina Bancells, Núria Casas-Vila, Núria Rovira-Graells, Carles Hernández-Ferrer, Juan Ramón González & Alfred Cortés

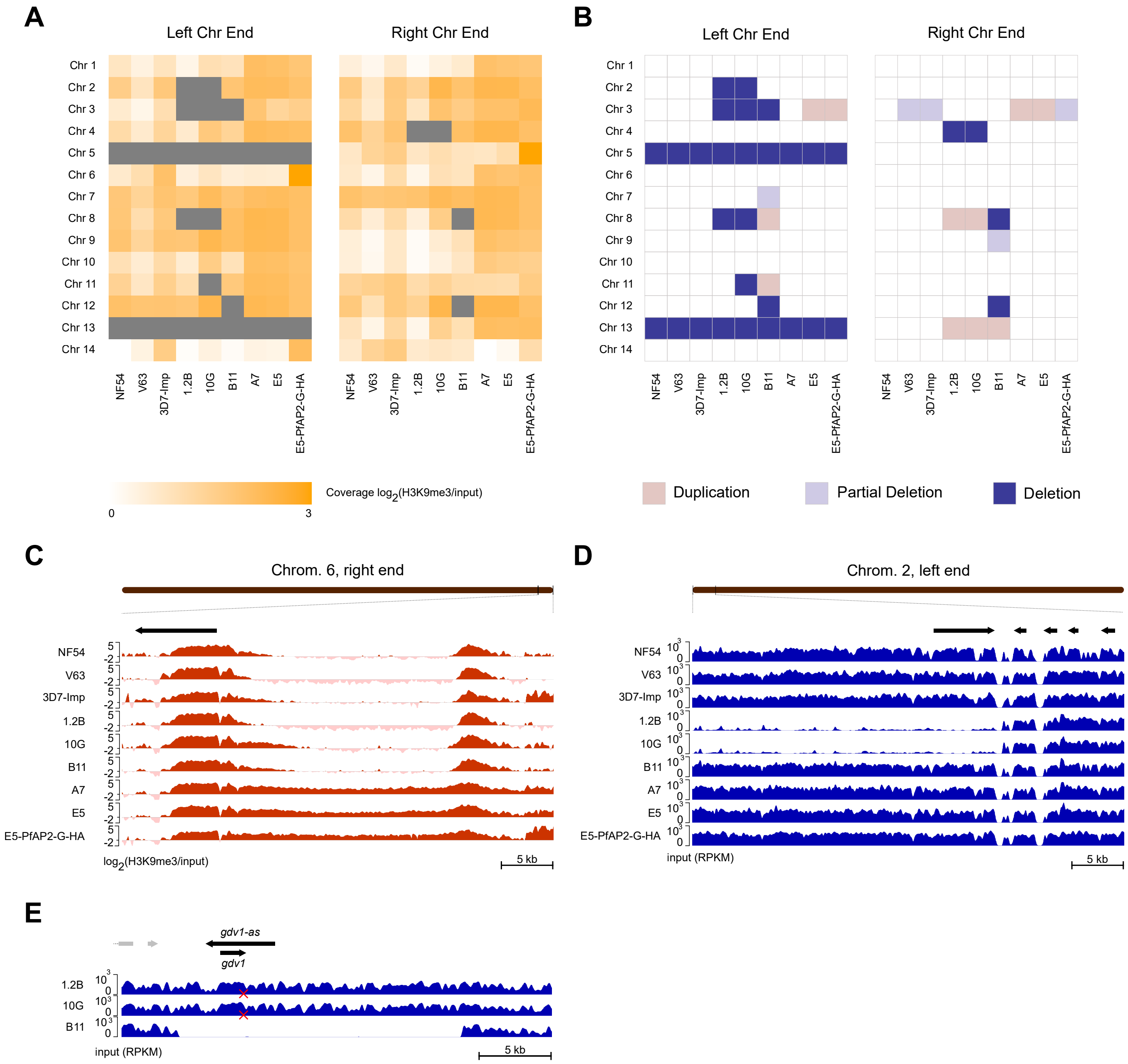

Fig. S1. Characterization of heterochromatin distribution, insertions and deletions at chromosome ends. (A) H3K9me3 coverage at chromosome ends, here defined as the subtelomeric non-coding regions from the most terminal gene to the telomere (right and left defined according to the orientation in PlasmoDB). Grey squares indicate presence of large deletions (>50% of the region analyzed) that preclude measuring heterochromatin levels. **(B)** Large deletions and duplications at chromosome ends (as defined in panel A). Chromosome ends with deletions that encompassed <30% of the region analyzed were marked as "partial deletion". **(C)** Representative example of differences between parasite lines in heterochromatin distribution at chromosome ends. **(D)** Representative example of deletions at chromosome ends. **(E)** Genomic alterations at the *gdv1* locus. A red cross indicates a SNP resulting in a premature STOP codon.

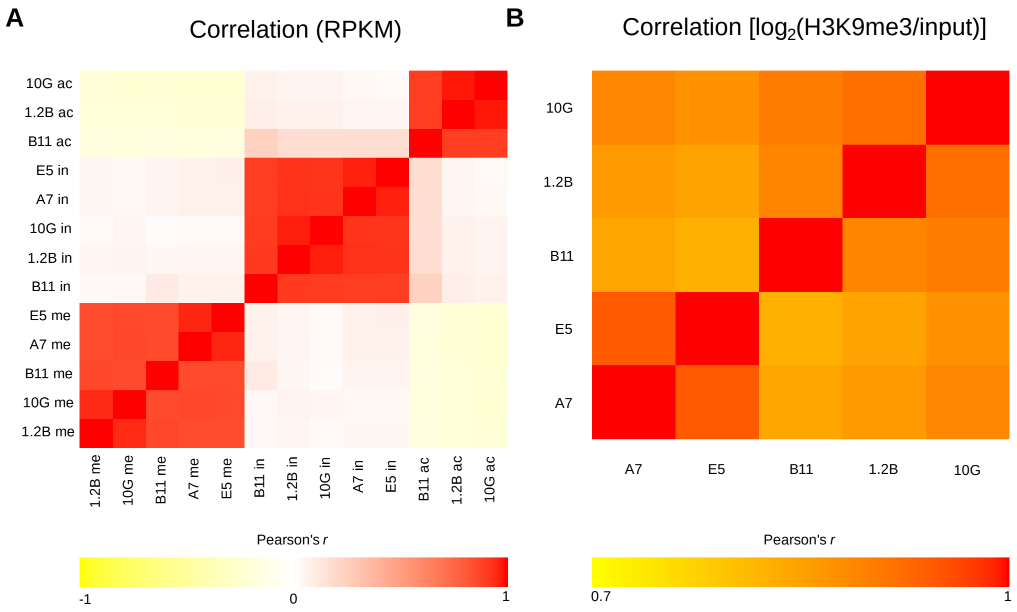

Fig. S2. Correlation between ChIP-Seq experiments. (A) Pearson correlation coefficient (*r*) between H3K9me3 (me), input (in) and H3K9ac (ac) ChIP-Seq signal (normalized coverage in RPKMs) for the five subclones of 3D7 genetic background analyzed. **(B)** Correlation between input-normalized H3K9me3 ChIP-Seq coverage for the five subclones.

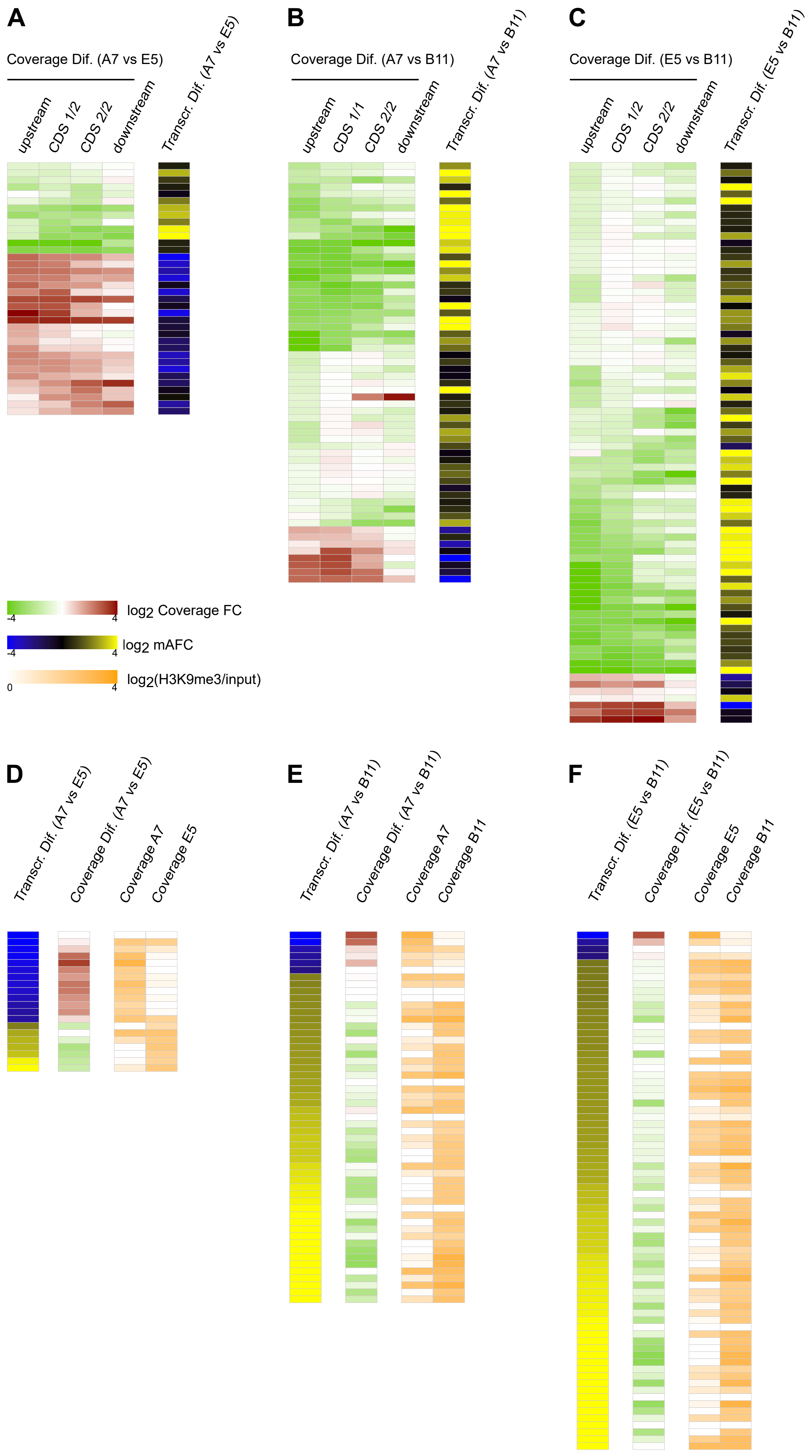

A
Coverage Dif. (A7 vs E5)
upstream
CDS 1/2
CDS 2/2
downstream
Transcr. Dif. (A7 vs E5)
B
Coverage Dif. (A7 vs B11)
upstream
CDS 1/1
CDS 2/2
downstream
Transcr. Dif. (A7 vs B11)
C
Coverage Dif. (E5 vs B11)
upstream
CDS 1/2
CDS 2/2
downstream
Transcr. Dif. (E5 vs B11)
-4
4
log2 Coverage FC
-4
4
log2 mAFC
0
4
log2(H3K9me3/input)
D
Transcr. Dif. (A7 vs E5)
Coverage Dif. (A7 vs E5)
Coverage A7
Coverage E5
E
Transcr. Dif. (A7 vs B11)
Coverage Dif. (A7 vs B11)
Coverage A7
Coverage B11
F
Transcr. Dif. (E5 vs B11)
Coverage Dif. (E5 vs B11)
Coverage E5
Coverage B11

Fig. S3. Association between H3K9me3 coverage differences and transcriptional differences. Results for subclones A7, E5 and B11 are shown (see main Fig. 3 for 10G and 1.2B). **(A-C)** Transcriptional differences in genes overlapping (-1,000 to +500 bp relative to the ATG) an H3K9me3 differential peak in the pairwise comparisons between A7 and E5 (A), A7 and B11 (B) or E5 and B11 (C). The $\log_2$ of the input-normalized H3K9me3 coverage fold-change (FC) is shown for the upstream region (1,000 bp before ATG), first or second half of the coding sequence (CDS1/2 or CDS2/2, respectively) and downstream region (1,000 bp after the STOP codon). Transcriptional differences are expressed as the $\log_2$ of the mAFC. **(D-F)** Heterochromatin levels in genes differentially expressed in each of the pairwise comparisons (mAFC >4). Transcriptional differences are shown as in panels A-C. H3K9me3 coverage ($\log_2$ of input-normalized H3K9me3 coverage) and $\log_2$ of coverage FC between A7 and E5 (D), A7 and B11 (E) or E5 and B11 (F) are shown for the region from -1,000 to +500 bp relative to the ATG. The values presented in this figure are provided in Data set S2.

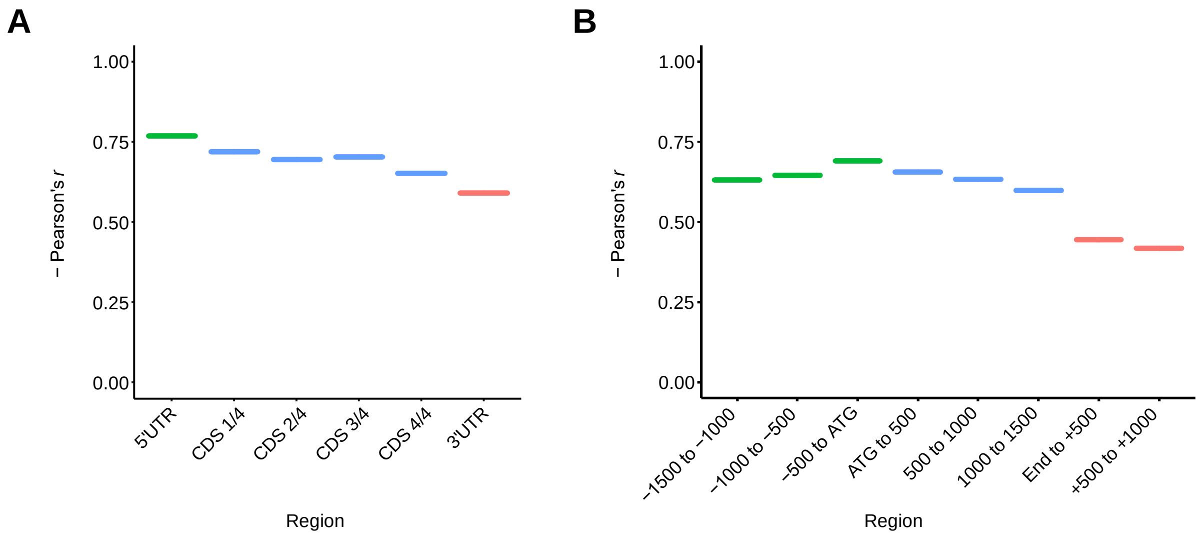

Fig. S4. Correlation between heterochromatin coverage and transcript levels at different positions of clonally variant loci. Correlation (Pearson's correlation coefficient, *r*) between input-normalized H3K9me3 coverage at different positions and transcript levels mAFC for genes with a mAFC >2 in any of the pairwise comparisons between 10G and 1.2B or among A7, E5 and B11. **(A)** H3K9me3 coverage was calculated for intervals of variable length corresponding to the 5'UTR (as annotated in PlasmoDB v52), coding sequence (CDS, divided in four different regions) and 3'UTR (as annotated in PlasmoDB v52). **(B)** H3K9me3 coverage was calculated for fixed length 500 bp regions relative to the start (ATG) or STOP (End) codon.

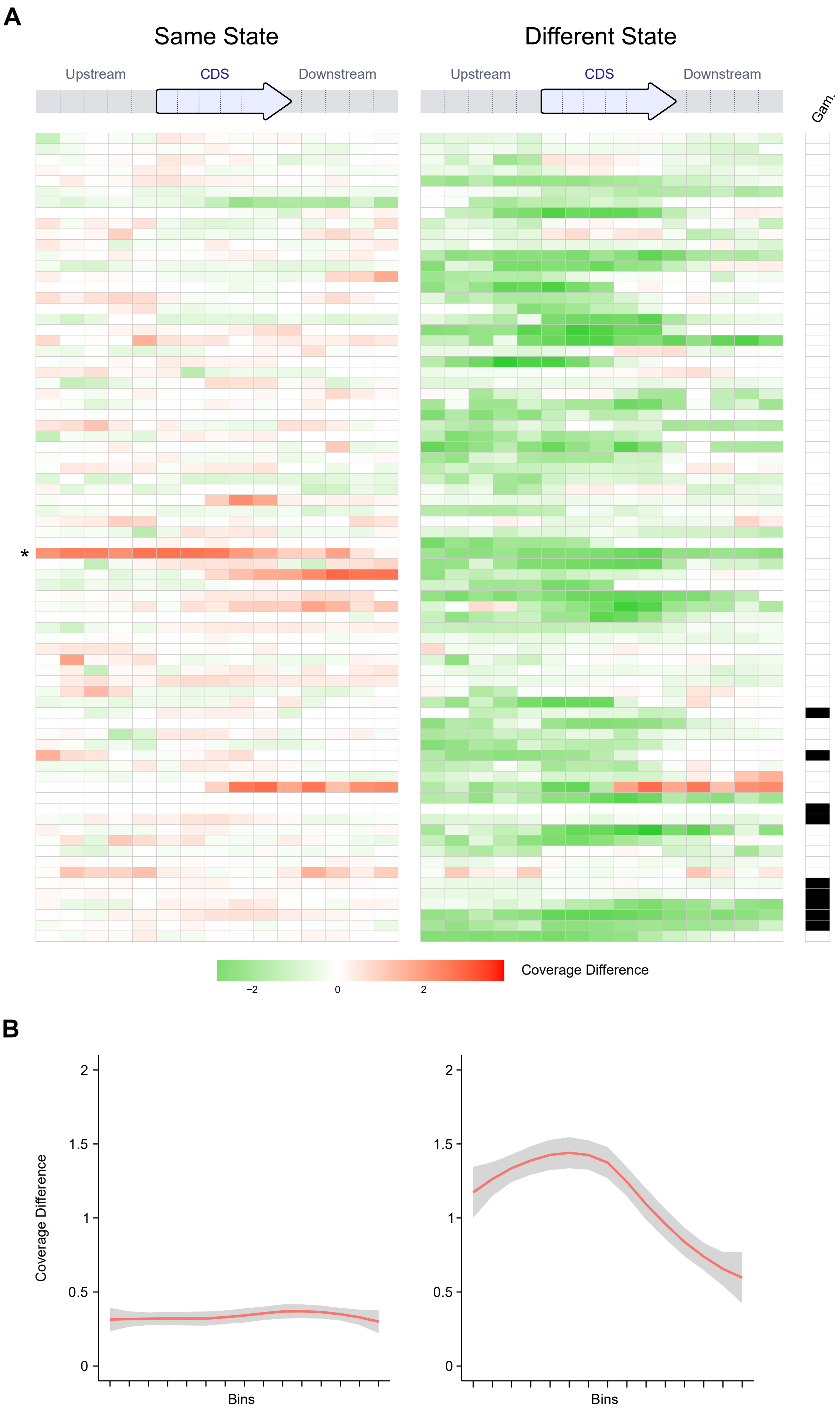
A
Same State
Different State
Upstream
CDS
Downstream
Upstream
CDS
Downstream
Gam.
*
−2
0
2
Coverage Difference
B
Coverage Difference
2
1.5
1
0.5
0
Bins
2
1.5
1
0.5
0
Bins

Fig. S5. Heterochromatin differences at differentially expressed genes between pairs of subclones in which the gene is in the same transcriptional state in the two subclones or in a different state. (A) H3K9me3 coverage differences at genes differentially expressed among the 10G, 1.2B, A7, E5 and B11 subclones. Differentially expressed genes were selected as in main Fig. 4, but only genes that were in the same state (active or silenced) in at least two subclones and in the opposite state in at least one subclone, according to the classification in Data set S6, were included. The coverage difference is shown as in main Fig. 4 but using only the 15 bins corresponding to the upstream sequence, coding sequence (CDS) and downstream sequence. In the left panel, H3K9me3 coverage differences are shown for two randomly-selected subclones that had the gene in the same state (active-active or silenced-silenced). In the right panel, H3K9me3 coverage differences are shown for two randomly-selected subclones that had the gene in a different state (active-silenced, coverage difference in the active vs the silenced state). The gene marked with an asterisk (PF3D7_0800800), which showed large H3K9me3 coverage differences in the upstream and coding regions between two subclones in which the gene was classified as being in the same transcriptional state (silenced), likely corresponds to an incorrect automatic classification (borderline values). Visual inspection revealed that this gene was classified as "silenced CVG" in 1.2B because it did not pass the threshold for differential expression in the classification algorithm [$\log_2$(mAFC): 0.9; threshold: 1], but the expression time course analysis showed consistently higher expression in 1.2B than in 10G, suggesting that actually it is likely an "active CVG" in 1.2B. The values presented in this figure are provided in Data set S2. **(B)** Loess regression plots (shades are 95% confidence intervals) for the absolute value of the data in panel A showing the difference in H3K9me3 coverage between subclones in which a gene is in the same transcriptional state (left) or in a different transcriptional state (right).

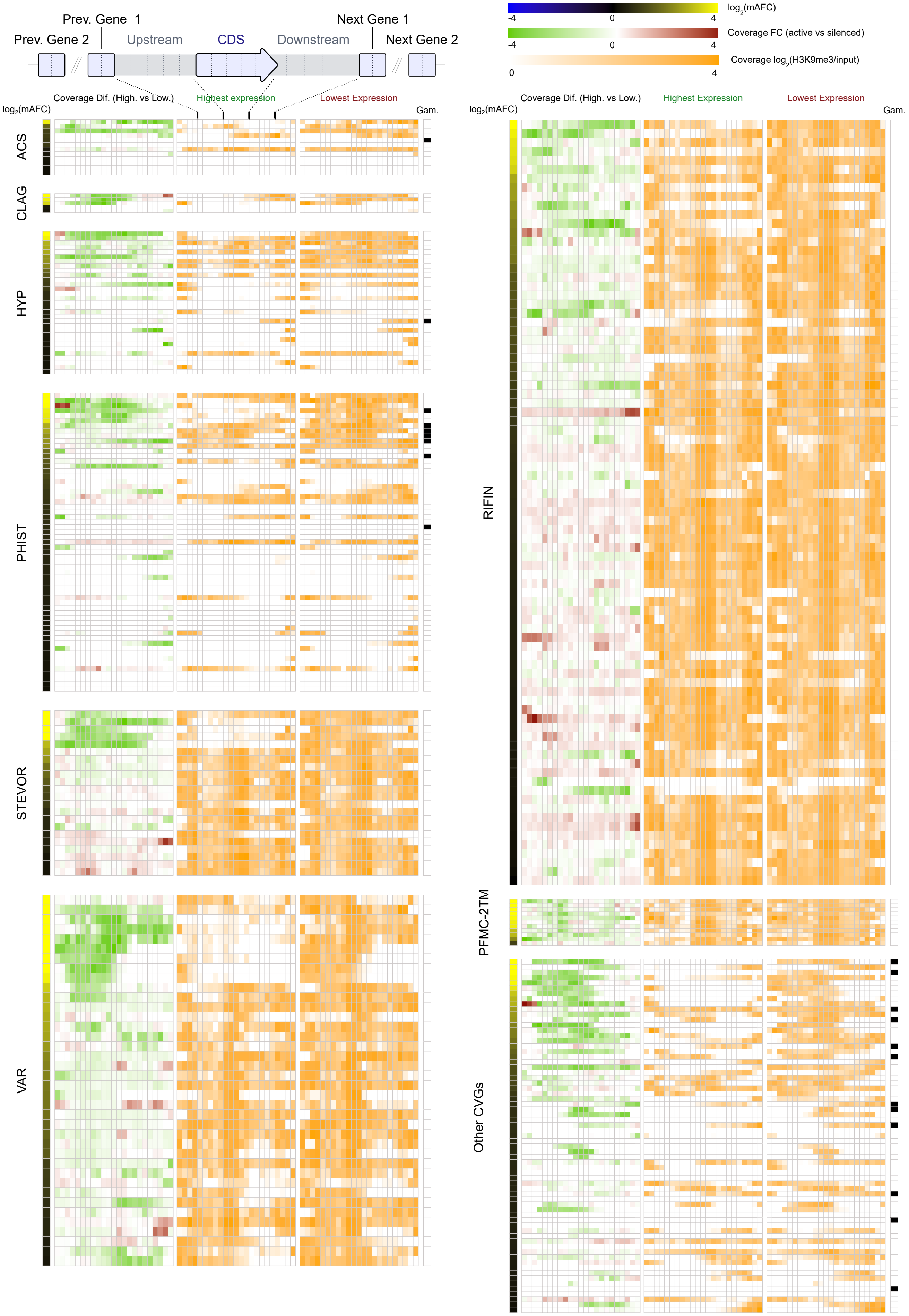

Prev. Gene 1
Prev. Gene 2
Upstream
CDS
Downstream
Next Gene 1
Next Gene 2
log2(mAFC)
-4
0
4
Coverage FC (active vs silenced)
-4
0
4
Coverage log2(H3K9me3/input)
0
4
Coverage Dif. (High. vs Low.)
Highest expression
Lowest Expression
Gam.
log2(mAFC)
ACS
CLAG
HYP
PHIST
STEVOR
VAR
RIFIN
PFMC-2TM
Other CVGs

Fig. S6. Distribution of heterochromatin in different CVG families. Distribution of H3K9me3 coverage at all genes of the main CVG families, regardless of whether or not a gene is differentially expressed among the 10G, 1.2B, A7, E5 and B11 subclones. For each gene, the H3K9me3 coverage for the subclone with highest expression and the subclone with lowest expression (selected as in main Fig. 4) is shown, together with the difference between the two. H3K9me3 coverage is shown for 23 bins spanning the upstream sequence until the previous gene, coding sequence (CDS), downstream sequence until the next gene and the two upstream and downstream neighbor genes, as in Fig. 4. The transcriptional difference [log_2(mAFC)] between the subclone with highest expression and the subclone with lowest expression (or 3D7-B instead of A7, E5 or B11 when the largest difference was between 10G or 1.2B and 3D7-B) is shown. Genes within each family are ordered by transcriptional difference. The column at the right indicates whether a gene is a known gametocyte marker. Information for the genes appearing in this figure is provided in Data set S2.

A

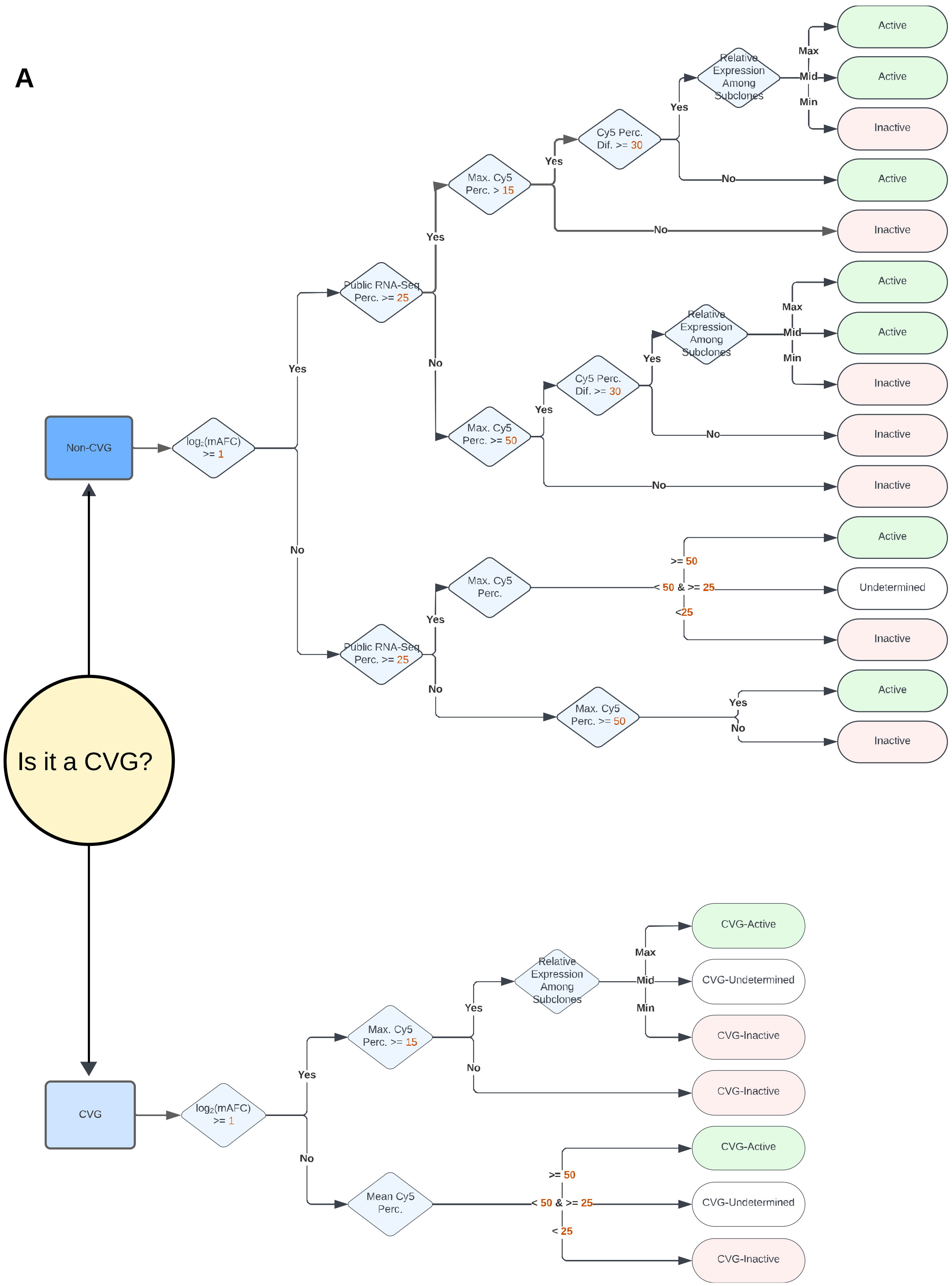

Is it a CVG?
Non-CVG
log2(mAFC) >= 1
Yes
No
Public RNA-Seq Perc. >= 25
Yes
No
Max. Cy5 Perc. > 15
Yes
No
Cy5 Perc. Dif. >= 30
Yes
No
Relative Expression Among Subclones
Max
Mid
Min
Active
Active
Inactive
Active
Inactive
Max. Cy5 Perc. >= 50
Yes
No
Cy5 Perc. Dif. >= 30
Yes
No
Relative Expression Among Subclones
Max
Mid
Min
Active
Active
Inactive
Inactive
Inactive
Public RNA-Seq Perc. >= 25
Yes
No
Max. Cy5 Perc.
>= 50
< 50 & >= 25
<25
Active
Undetermined
Inactive
Max. Cy5 Perc. >= 50
Yes
No
Active
Inactive
CVG
log2(mAFC) >= 1
Yes
No
Max. Cy5 Perc. >= 15
Yes
No
Relative Expression Among Subclones
Max
Mid
Min
CVG-Active
CVG-Undetermined
CVG-Inactive
CVG-Inactive
Mean Cy5 Perc.
>= 50
< 50 & >= 25
< 25
CVG-Active
CVG-Undetermined
CVG-Inactive

B

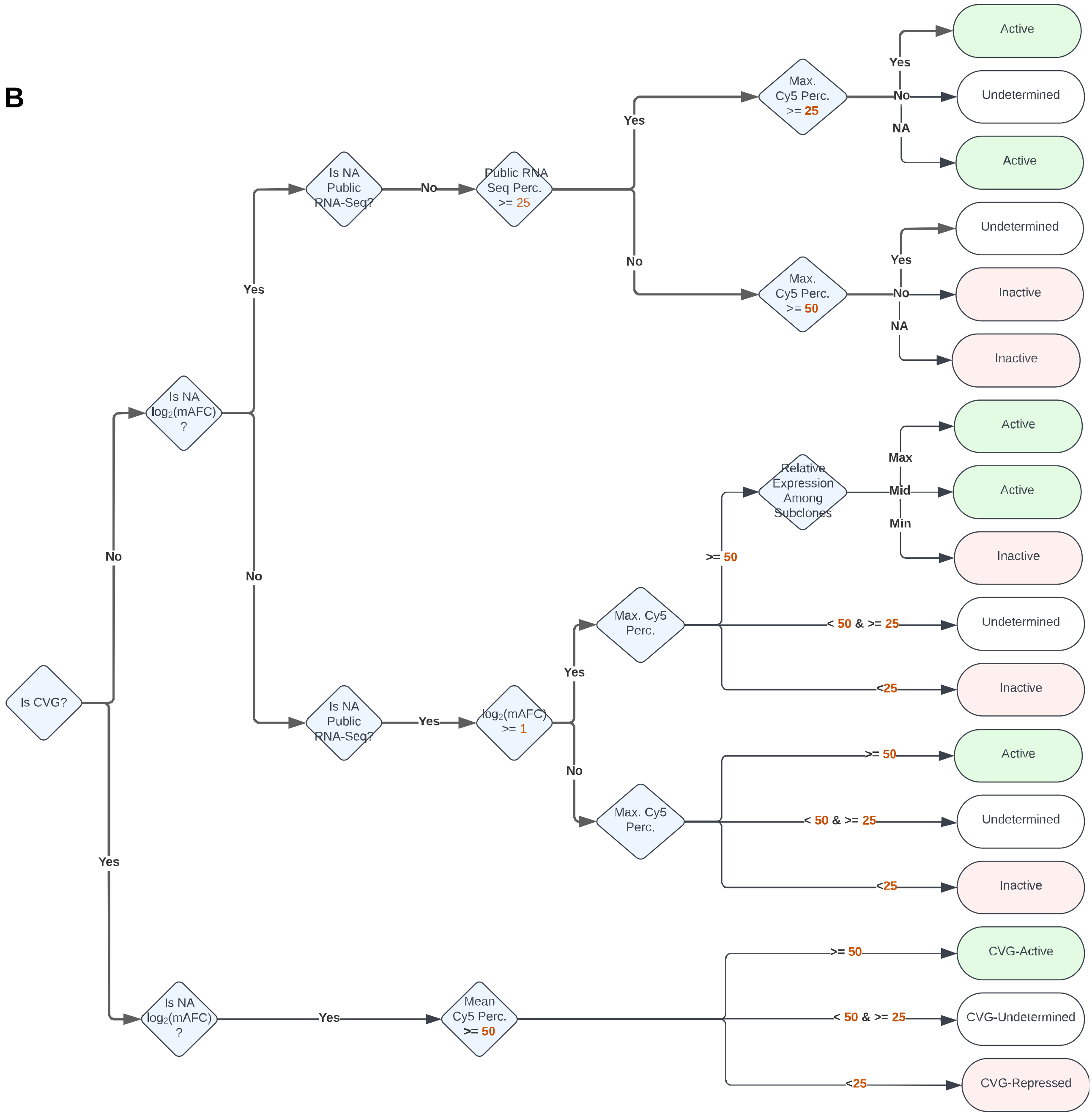

Fig. S7. Algorithm used to classify genes according to their transcriptional state in the subclones analyzed in this study. The algorithm was used to classify genes in the 1.2B, 10G, A7, E5 and B11 subclones. Separate decision trees are shown for genes with values for all classifying variables (A) and for genes with missing values (B). The state of each gene in each of the five subclone is shown in Data set S6. **(A)** Classification tree for genes without missing values. Genes were initially classified as CVGs or non-CVGs based on a previously published list of CVGs (Pickford et al., mBio 2021, PMID: 34340541). Then, 5 different classification criteria where used in the order indicated in the tree: (i) differential expression among the five subclones (log_2(mAFC) >1 in at least one pairwise comparison); (ii) expression level percentile ≥25 in at least one of four selected publicly available RNA-Seq datasets (Public RNA-Seq Perc.) (Bartfai et al. PLoS Pathog. 2010, PMID: 21187892; Otto et al., Mol. Microbiol. 2010, PMID: 20141604; Kensche et al.Nucleic Acids Res. 2016, PMID: 26578577; Toenhake et al., Cell Host Microbe 2018, PMID: 29649445); (iii) expression level in the microarray analysis presented here using the average of the two highest Cy5 percentile values (among all time points), either the maximum among the subclones (Max Cy5 Perc.) or the mean (Mean Cy5 Perc.), with different threshold values; (iv) the maximum difference in Cy5 percentile values between

any two subclones (Cy5 Perc. Dif.); (v) the relative expression between subclones. For this, the subclone with the highest expression value (using average normalized Cy5/Cy3 across the time interval with highest expression) was always classified as “max” and the subclone with lowest expression as “min”. For the remaining subclones, an upper threshold and a lower threshold where defined by dividing the distance between “max” and “min” in three equal intervals. The remaining subclones where classified as “max” if above the upper threshold, as “mid” if between the upper and lower thresholds and “min” if under the lower threshold. Based on these criteria and the decision tree, in each of the five subclones non-CVGs were classified as *Active*, *Inactive* or *Undetermined* and CVGs were classified as *CVG-active*, *CVG-silenced* or *CVG-Undetermined*. **(B)** Classification tree for genes with missing values. The classification criteria were the same as in panel A, but the decision tree structure was modified to account for the missing values.
